# New *q*-Analogues of Van Hamme’s (E.2) Supercongruence and of a Supercongruence by Swisher

**DOI:** 10.1007/s00025-023-01885-8

**Published:** 2023-03-21

**Authors:** Victor J. W. Guo, Michael J. Schlosser

**Affiliations:** 1 School of Mathematics and Statistics, Huaiyin Normal University, Huai’an, 223300 Jiangsu People’s Republic of China; 2 Fakultät für Mathematik, Universität Wien, Oskar-Morgenstern-Platz 1, A-1090 Vienna, Austria

**Keywords:** Basic hypergeometric series, supercongruences, *q*-congruences, cyclotomic polynomial, ${}_6\phi _5$ summation, Primary 33D15, Secondary 11A07

## Abstract

In this paper, a couple of *q*-supercongruences for truncated basic hypergeometric series are proved, most of them modulo the cube of a cyclotomic polynomial. One of these results is a new *q*-analogue of the (E.2) supercongruence by Van Hamme, another one is a new *q*-analogue of a supercongruence by Swisher, while the other results are closely related *q*-supercongruences. The proofs make use of special cases of a very-well-poised ${}_6\phi _5$ summation. In addition, the proofs utilize the method of creative microscoping (which is a method recently introduced by the first author in collaboration with Wadim Zudilin), and the Chinese remainder theorem for coprime polynomials.

## Introduction

In 1997, Van Hamme [24] presented 13 remarkable supercongruences corresponding to Ramanujan’s or to Ramanujan-like formulas for $1/\pi$. For instance, the two infinite series expansions

$$\begin{aligned}&\sum _{k=0}^\infty (-1)^k(4k+1)\frac{(\frac{1}{2})_k^3}{k!^3} =\frac{2}{\pi }, \\&\sum _{k=0}^\infty (-1)^k(6k+1)\frac{(\frac{1}{3})_k^3}{k!^3} =\frac{3\sqrt{3}}{2\pi }, \end{aligned}$$

correspond to the following two supercongruences for truncated hypergeometric series:

1.1 $$\begin{aligned} \sum _{k=0}^{(p-1)/2} (-1)^k(4k+1)\frac{(\frac{1}{2})_k^3}{k!^3}&\equiv (-1)^{(p-1)/2}p\pmod {p^3}, \end{aligned}$$

1.2 $$\begin{aligned} \sum _{k=0}^{(p-1)/3} (-1)^k(6k+1)\frac{(\frac{1}{3})_k^3}{k!^3}&\equiv p\pmod {p^3},\quad \text {for}\quad p\equiv 1\pmod {3}, \end{aligned}$$

where *p* is an odd prime, and $(a)_n=a(a+1)\cdots (a+n-1)$ denotes the Pochhammer symbol. The supercongruence (1.1) was first proved by Mortenson [18] using a technical evaluation of gamma functions, and later reproved by Zudilin [28] and Long [16]. Swisher [23] employed Long’s method to prove four supercongruences of Van Hamme, including (1.2) (i.e., the (E.2) supercongruence in [24]). He [11] also gave a generalization of (1.2). In 2016, Osburn and Zudilin [21] confirmed the last supercongruence conjecture of Van Hamme.

During the past few years, *q*-analogues of supercongruences have been investigated by many authors (see, for example, [3–10, 12–15, 19, 20, 22, 25–27, 29]). In particular, the first author [3, 4] gave *q*-analogues of (1.1) and (1.2) as follows: for any odd integer *n*,

$$\begin{aligned} \sum _{k=0}^{(n-1)/2}(-1)^k [4k+1]\frac{(q;q^2)_k^3}{(q^2;q^2)_k^3}q^{k^2} \equiv (-1)^{(n-1)/2}q^{(n-1)^2/4} [n]\pmod {[n]\Phi _n(q)^2}, \end{aligned}$$

and for any positive integer *n* with $n\equiv 1\pmod {3}$,

$$\begin{aligned} \sum _{k=0}^{(n-1)/3}(-1)^k[6k+1]\frac{(q;q^3)_k^3}{(q^3;q^3)_k^3}q^{(3k^2+k)/2} \equiv&(-1)^{(n-1)/3}q^{(n-1)(n-2)/6}[n] \\&\pmod {[n]\Phi _n(q)^2}. \end{aligned}$$

Here and in what follows, $(a;q)_n=(1-a)(1-aq)\cdots (1-aq^{n-1})$ is the *q*-*shifted factorial*, $[n]=[n]_q=(1-q^n)/(1-q)$ is the *q*-*integer*, and $\Phi _n(q)$ denotes the *n*-th *cyclotomic polynomial* in *q*, i.e.,

$$\begin{aligned} \Phi _n(q)=\prod _{\begin{array}{c} 1\leqslant k\leqslant n\\ \gcd (k,n)=1 \end{array}}(q-\zeta ^k), \end{aligned}$$

where $\zeta$ is an *n*-th primitive root of unity. The first author and Zudilin [10, Theorem 3.5 with $r=1$] also gave another *q*-analogue of (1.2): for any positive integer *n* with $n\equiv 1\pmod {6}$,

1.3 $$\begin{aligned} \sum _{k=0}^{(n-1)/3}(-1)^k[6k+1]_{q^2}\frac{(q^2;q^6)_k^3 (-q^3;q^6)_k}{(q^6;q^6)_k^3 (-q^5;q^6)_k}q^k \equiv q^{1-n} [n]_{q^2} \pmod {[n]\Phi _n(q)^2}. \end{aligned}$$

One of the aims of this paper is to establish the following new *q*-analogue of (1.2).

### Theorem 1.1

Let $n\equiv 1\pmod {6}$ be a positive integer. Then

1.4 $$\begin{aligned} \sum _{k=0}^{M} (-1)^k[6k+1]\frac{(q;q^3)_k^3}{(q^3;q^3)_k^3} \equiv q^{2(1-n)/3}[n]\frac{(-q^3;q^3)_{(n-1)/3}}{(-q^2;q^3)_{(n-1)/3}} \pmod {[n]\Phi _n(q)^2}, \end{aligned}$$

where $$M=(n-1)/3$$ or $$M=n-1$$.

We shall also give the following similar result.

### Theorem 1.2

Let $n\equiv 1\pmod {3}$ be a positive integer. Then

1.5 $$\begin{aligned} \sum _{k=0}^{M} [6k+1]\frac{(q;q^3)_k^3}{(q^3;q^3)_k^3} \equiv q^{2(1-n)/3}[n]\frac{(q^3;q^3)_{(n-1)/3}}{(q^2;q^3)_{(n-1)/3}} \pmod {\Phi _n(q)^3}, \end{aligned}$$

where $$M=(n-1)/3$$ or $$M=n-1$$.

Note that the supercongruence (1.5) does not hold modulo $[n]\Phi _n(q)^2$ in general, even for $n\equiv 1\pmod {6}$. We take this opportunity to point out that Theorems 1 and 2 in [8] only hold modulo $\Phi _n(q)^3$ and $\Phi _n(q)^2$, respectively, but do not hold modulo [*n*], since Lemma 3 in [8] is not true (it only holds for even integers *d*).

Swisher [23] also proved that, for any prime $p\equiv 2\pmod {3}$,

1.6 $$\begin{aligned} \sum _{k=0}^{(2p-1)/3} (-1)^k(6k+1)\frac{(\frac{1}{3})_k^3}{k!^3}&\equiv -2p\pmod {p^3}. \end{aligned}$$

A *q*-analogue of (1.6) was given by the first author [4, Theorem 1.5 with $(d,r)=(3,1)$]: for any positive integer $n\equiv 2\pmod {3}$,

$$\begin{aligned}&\sum _{k=0}^{(2n-1)/3}(-1)^kq^{(3k^2+k)/2}[6k+1]\frac{(q;q^3)_k^3}{(q^3;q^3)_k^3} \equiv -[2n]q^{(n-1)(2n-1)/3} \\&\pmod {[n]\Phi _n(q)^2}. \end{aligned}$$

In this paper, we shall give a new *q*-analogue of (1.6).

### Theorem 1.3

Let $n\equiv 5\pmod {6}$ be a positive integer. Then

1.7 $$\begin{aligned} \sum _{k=0}^{M} (-1)^k[6k+1]\frac{(q;q^3)_k^3}{(q^3;q^3)_k^3} \equiv -q^{2(1-2n)/3}[2n]\frac{(-q^3;q^3)_{(2n-1)/3}}{(-q^2;q^3)_{(2n-1)/3}} \pmod {[n]\Phi _n(q)^2}, \end{aligned}$$

where $$M=(2n-1)/3$$ or $$M=n-1$$.

Similarly, we have the following result.

### Theorem 1.4

Let $n\equiv 2\pmod {3}$ be a positive integer. Then

1.8 $$\begin{aligned} \sum _{k=0}^{M} [6k+1]\frac{(q;q^3)_k^3}{(q^3;q^3)_k^3} \equiv q^{2(1-2n)/3}[2n]\frac{(q^3;q^3)_{(2n-1)/3}}{(q^2;q^3)_{(2n-1)/3}} \pmod {\Phi _n(q)^2}, \end{aligned}$$

where $$M=(2n-1)/3$$ or $$M=n-1$$.

Note that the *q*-supercongruence (1.8) does not hold modulo $\Phi _n(q)^3$ for $n>2$. We shall prove Theorems 1.1, 1.2, and 1.3 modulo $\Phi _n(q)^3$ and Theorem 1.4 by using a summation for a very-well-poised ${}_6\phi _5$ series and the ‘creative microscoping’ method introduced by the first author in collaboration with Zudilin [9]. The proof of Theorems 1.1 and 1.3 also requires the use of a lemma previously given by the present authors.

From Theorems 1.2 and 1.4, we can deduce the following supercongruences.

### Corollary 1.5

Let $p\equiv 1\pmod {3}$ be a prime. Then

1.9 $$\begin{aligned} \sum _{k=0}^{(p-1)/3} (6k+1)\frac{(\frac{1}{3})_k^3}{k!^3} \equiv p\Gamma _p(\tfrac{2}{3})^3\pmod {p^3}, \end{aligned}$$

where $\Gamma _p(x)$ denotes the *p*-adic Gamma function.

### Corollary 1.6

Let $p\equiv 2\pmod {3}$ be an odd prime. Then

$$\begin{aligned} \sum _{k=0}^{(2p-1)/3} (6k+1)\frac{(\frac{1}{3})_k^3}{k!^3} \equiv -6\Gamma _p(\tfrac{2}{3})^3\pmod {p^2}. \end{aligned}$$

## Proof of Theorem 1.1

We first give the following result, which is due to the present authors [6, Lemma 2.1].

### Lemma 2.1

Let *d*, *m* and *n* be positive integers with $m\leqslant n-1$. Let *r* be an integer satisfying $dm\equiv -r\pmod {n}$. Then, for $0\leqslant k\leqslant m$ and any indeterminate *a*, we have

$$\begin{aligned} \frac{(aq^r;q^d)_{m-k}}{(q^d/a;q^d)_{m-k}} \equiv (-a)^{m-2k}\frac{(aq^r;q^d)_k}{(q^d/a;q^d)_k} q^{m(dm-d+2r)/2+(d-r)k} \pmod {\Phi _n(q)}. \end{aligned}$$

If $\gcd (d,n)=1$, then the above *q*-congruence also holds for $a=1$.

We also need the following result to prove the truth of (1.4) modulo [*n*].

### Lemma 2.2

Let *n* be a positive integer coprime with 6, and let *a* be an indeterminate. Then

2.1 $$\begin{aligned}&\sum _{k=0}^{m}(-1)^k[6k+1]\frac{(aq;q^3)_k(q/a;q^3)_k(q;q^3)_k }{(aq^3;q^3)_k (q^3/a;q^3)_k (q^3;q^3)_k } \equiv 0\pmod {[n]}, \end{aligned}$$

2.2 $$\begin{aligned}&\sum _{k=0}^{n-1}(-1)^k[6k+1]\frac{(aq;q^3)_k(q/a;q^3)_k(q;q^3)_k }{(aq^3;q^3)_k (q^3/a;q^3)_k (q^3;q^3)_k } \equiv 0\pmod {[n]}, \end{aligned}$$

where $m=(n-1)/3$ if $n\equiv 1\pmod {6}$, and $m=(2n-1)/3$ if $n\equiv 5\pmod {6}$.

### Proof

Clearly, Lemma 2.2 is true for $n=1$. We now assume that $n>1$. By Lemma 2.1, we can easily deduce that the *k*-th and $(m-k)$-th terms on the left-hand side of (2.1) cancel each other modulo $\Phi _n(q)$, i.e.,

$$\begin{aligned}&(-1)^{m-k}\frac{[6(m-k)+1](aq;q^3)_{m-k}(q/a;q^3)_{m-k}(q;q^3)_{m-k} }{(aq^3;q^3)_{m-k} (q^3/a;q^3)_{m-k} (q^3;q^3)_{m-k} } \\&\qquad \equiv -(-1)^k[6k+1]\frac{(aq;q^3)_k(q/a;q^3)_k(q;q^3)_k }{(aq^3;q^3)_k (q^3/a;q^3)_k (q^3;q^3)_k }\pmod {\Phi _n(q)}. \end{aligned}$$

Thus, we have proved that the *q*-congruence (2.1) holds modulo $\Phi _n(q)$. Since the numerator contains the factor $(q;q^3)_k$, it is easy to see that the *k*-th summand in (2.2) is congruent to 0 modulo $\Phi _n(q)$ for $m< k\leqslant n-1$. This proves the *q*-congruence (2.2) modulo $\Phi _n(q)$.

Now we can prove (2.1) and (2.2) modulo [*n*]. Let $\zeta \ne 1$ be an *n*-th root of unity, not necessarily primitive. In other words, $\zeta$ is a primitive root of unity of degree *s* satisfying $s\mid n$ and $s>1$. Let $c_q(k)$ stand for the *k*-th term on the left-hand side of (2.2), i.e.,

$$\begin{aligned} c_q(k) =(-1)^k[6k+1]\frac{(aq;q^3)_k(q/a;q^3)_k(q;q^3)_k }{(aq^3;q^3)_k (q^3/a;q^3)_k (q^3;q^3)_k }. \end{aligned}$$

Taking $n=s$ in the *q*-congruences (2.1) and (2.2) modulo $\Phi _n(q)$, we get

$$\begin{aligned} \sum _{k=0}^{s_1}c_\zeta (k)=\sum _{k=0}^{s-1}c_\zeta (k)=0, \end{aligned}$$

where $s_1=(s-1)/3$ if $s\equiv 1\pmod {6}$, and $s_1=(2s-1)/3$ if $s\equiv 5\pmod {6}$. It is not difficult to see that

$$\begin{aligned} \lim _{q\rightarrow \zeta }\frac{c_q(\ell s+k)}{c_q(\ell s)} =\frac{c_\zeta (\ell s+k)}{c_\zeta (\ell s)} =c_\zeta (k). \end{aligned}$$

Therefore,

2.3 $$\begin{aligned} \sum _{k=0}^{n-1}c_\zeta (k)=\sum _{\ell =0}^{n/s-1} \sum _{k=0}^{s-1}c_\zeta (\ell s+k) =\sum _{\ell =0}^{n/s-1}c_\zeta (\ell s) \sum _{k=0}^{s-1}c_\zeta (k)=0, \end{aligned}$$

and

$$\begin{aligned} \sum _{k=0}^{m}c_\zeta (k) =\sum _{\ell =0}^{(m-s_1)/s-1} c_\zeta (\ell s)\sum _{k=0}^{s-1}c_\zeta (k) +c_\zeta (m-s_1)\sum _{k=0}^{s_1}c_\zeta (k)=0. \end{aligned}$$

This proves that both of the sums $\sum _{k=0}^{n-1}c_q(k)$ and $\sum _{k=0}^{m}c_q(k)$ are divisible by $\Phi _s(q)$ for any divisor $s>1$ of *n*. Since

$$\begin{aligned} \prod _{s\mid n,\, s>1}\Phi _s(q)=[n], \end{aligned}$$

we complete the proof of (2.1) and (2.2). $\square$

Like most of the *q*-supercongruences in [9], we have the following parametric generalization of Theorem 1.1.

### Theorem 2.3

Let $n\equiv 1\pmod {6}$ be a positive integer. Then, modulo $[n](1-aq^n)(a-q^n)$,

2.4 $$\begin{aligned} \sum _{k=0}^{M} (-1)^k[6k+1]\frac{(aq;q^3)_k(q/a;q^3)_k(q;q^3)_k }{(aq^3;q^3)_k (q^3/a;q^3)_k (q^3;q^3)_k } \equiv q^{2(n-1)/3}[n]\frac{(-q^3;q^3)_{(n-1)/3}}{(-q^2;q^3)_{(n-1)/3}}, \end{aligned}$$

where $$M=(n-1)/3$$ or $$M=n-1$$.

### Proof

We start with the following summation for a very-well-poised ${}_6\phi _5$ series (see [2, Appendix (II.20)]):

2.5 $$\begin{aligned}&\sum _{k=0}^\infty \frac{(1-aq^{2k})(a;q)_k(b;q)_k(c;q)_k(d;q)_k}{(1-a)(q;q)_k(aq/b;q)_k(aq/c;q)_k(aq/d;q)_k}\biggl (\frac{aq}{bcd}\biggr )^k \nonumber \\&\quad =\frac{(aq;q)_\infty (aq/bc;q)_\infty (aq/bd;q)_\infty (aq/cd;q)_\infty }{(aq/b;q)_\infty (aq/c;q)_\infty (aq/d;q)_\infty (aq/bcd;q)_\infty }. \end{aligned}$$

(The infinite series in (2.5) converges for $|q|<1$ and $|aq/bcd|<1$.) Specializing (2.5) by letting $q\mapsto q^3$, $a=q$, $b=q^{1-n}$, $c=q^{1+n}$, and $d=-q^2$, we have

$$\begin{aligned}&\sum _{k=0}^{(n-1)/3} (-1)^k[6k+1]\frac{(q^{1-n};q^3)_k(q^{1+n};q^3)_k(q;q^3)_k }{(q^{3-n};q^3)_k (q^{3+n};q^3)_k (q^3;q^3)_k } \\&\quad \quad =\frac{(q^4;q^3)_{(n-1)/3} (-q^{1-n};q^3)_{(n-1)/3}}{(q^{3-n};q^3)_{(n-1)/3}(-q^2;q^3)_{(n-1)/3}} \\&\quad \quad =q^{2(1-n)/3}[n]\frac{(-q^3;q^3)_{(n-1)/3}}{(-q^2;q^3)_{(n-1)/3}}. \end{aligned}$$

This shows that both sides of (2.4) are equal for $a=q^{-n}$ and $a=q^n$. This means that the congruence (2.4) holds modulo $1-aq^n$ and $a-q^n$.

Moreover, by Lemma 2.2, the left-hand side of (2.4) is congruent to 0 modulo [*n*]. Since $1-q^n$ (*n* is odd) is relatively prime to $1+q^k$, we see that the right-hand side of (2.4) is also congruent to 0 modulo [*n*]. Noticing that $1-aq^n$, $a-q^n$, and [*n*] are pairwise coprime polynomials in *q*, we finish the proof of the theorem. $\square$

### Proof of Theorem 1.1

Since $(1-q^n)^2$ contains the factor $\Phi _n(q)^2$ and $(q^3;q^3)_M$ is coprime with $\Phi _n(q)$, letting $a=1$ in (2.4), we conclude that (1.4) is true modulo $\Phi _n(q)^3$. Note that Lemma 2.2 also holds for $a=1$. Namely, the *q*-congruence (1.4) is true modulo [*n*] and is therefore also true modulo $[n]\Phi _n(q)^2$. This completes the proof. $\square$

## Proof of Theorem 1.2

We first give the following parametric generalization of Theorem 1.2: for $n\equiv 1\pmod {3}$, modulo $\Phi _n(q)(1-aq^n)(a-q^n)$,

3.1 $$\begin{aligned} \sum _{k=0}^{M} [6k+1]\frac{(aq;q^3)_k(q/a;q^3)_k(q;q^3)_k }{(aq^3;q^3)_k (q^3/a;q^3)_k (q^3;q^3)_k } \equiv q^{2(n-1)/3}[n]\frac{(q^3;q^3)_{(n-1)/3}}{(q^2;q^3)_{(n-1)/3}},\nonumber \\ \end{aligned}$$

where $M=(n-1)/3$ or $M=n-1$. The proof of (3.1) is analogous to that of (2.4). This time, we make the substitutions $q\mapsto q^3$, $a=q$, $b=q^{1-n}$, $c=q^{1+n}$, and $d=q^2$ in (2.5) to obtain

$$\begin{aligned}&\sum _{k=0}^{(n-1)/3} [6k+1]\frac{(q^{1-n};q^3)_k(q^{1+n};q^3)_k(q;q^3)_k }{(q^{3-n};q^3)_k (q^{3+n};q^3)_k (q^3;q^3)_k } \\&\quad \quad =\frac{(q^4;q^3)_{(n-1)/3} (q^{1-n};q^3)_{(n-1)/3}}{(q^{3-n};q^3)_{(n-1)/3}(q^2;q^3)_{(n-1)/3}} \\&\quad \quad =q^{2(1-n)/3}[n]\frac{(q^3;q^3)_{(n-1)/3}}{(q^2;q^3)_{(n-1)/3}}. \end{aligned}$$

Thus, the two sides of (3.1) are equal for $a=q^{-n}$ and $a=q^n$. This means that the congruence (3.1) is true modulo $1-aq^n$ and $a-q^n$.

Moreover, by Lemma 2.1, for $m=(n-1)/3$ we can deduce that the *k*-th and $(m-k)$-th terms on the left-hand side of (3.1) cancel each other modulo $\Phi _n(q)$, i.e.,

$$\begin{aligned}&\frac{[6(m-k)+1](aq;q^3)_{m-k}(q/a;q^3)_{m-k}(q;q^3)_{m-k} }{(aq^3;q^3)_{m-k} (q^3/a;q^3)_{m-k} (q^3;q^3)_{m-k} } \\&\qquad \equiv -[6k+1]\frac{(aq;q^3)_k(q/a;q^3)_k(q;q^3)_k }{(aq^3;q^3)_k (q^3/a;q^3)_k (q^3;q^3)_k }\pmod {\Phi _n(q)}. \end{aligned}$$

(Note that we have utilized the fact that $q^{n/2}\equiv -1\pmod {\Phi _n(q)}$ for even *n*.) This proves (3.1) modulo $\Phi _n(q)$.

Finally, letting $a=1$ in (3.1), we arrive at the *q*-supercongruence (1.5).

## Proof of Theorems 1.3 and 1.4

### Proof of Theorem 1.3

We first give a parametric generalization of Theorem 1.4: for $n\equiv 5\pmod {6}$, modulo $[n](1-aq^{2n})(a-q^{2n})$,

4.1 $$\begin{aligned}&\sum _{k=0}^{M} (-1)^k[6k+1]\frac{(aq;q^3)_k(q/a;q^3)_k(q;q^3)_k }{(aq^3;q^3)_k (q^3/a;q^3)_k (q^3;q^3)_k } \equiv -q^{2(1-2n)/3}[2n]\nonumber \\&\quad \frac{(-q^3;q^3)_{(2n-1)/3}}{(-q^2;q^3)_{(2n-1)/3}}, \end{aligned}$$

where $M=(2n-1)/3$ or $n-1$. The proof of (4.1) is very similar to that of (2.4). Specializing (2.5) by $q\mapsto q^3$, $a=q$, $b=q^{1-2n}$, $c=q^{1+2n}$, and $d=-q^2$, we have

$$\begin{aligned}&\sum _{k=0}^{(2n-1)/3} (-1)^k[6k+1]\frac{(q^{1-2n};q^3)_k(q^{1+2n};q^3)_k(q;q^3)_k }{(q^{3-2n};q^3)_k (q^{3+2n};q^3)_k (q^3;q^3)_k } \\&\quad \quad =\frac{(q^4;q^3)_{(2n-1)/3} (-q^{1-2n};q^3)_{(2n-1)/3}}{(q^{3-2n};q^3)_{(2n-1)/3}(-q^2;q^3)_{(2n-1)/3}} \\&\quad \quad =-q^{2(1-2n)/3}[2n]\frac{(-q^3;q^3)_{(2n-1)/3}}{(-q^2;q^3)_{(2n-1)/3}}. \end{aligned}$$

This proves the congruence (4.1) modulo $1-aq^{2n}$ and $a-q^{2n}$. Moreover, the proof of (4.1) modulo [*n*] follows from Lemma 2.2. $\square$

Finally, taking $a=1$ in (4.1), we arrive at the desired *q*-supercongruence (1.7).

### Proof of Theorem 1.4

We have the following congruence with a parameter *a*: for $n\equiv 5\pmod {6}$, modulo $(1-aq^{2n})(a-q^{2n})$,

4.2 $$\begin{aligned} \sum _{k=0}^{M} [6k+1]\frac{(aq;q^3)_k(q/a;q^3)_k(q;q^3)_k }{(aq^3;q^3)_k (q^3/a;q^3)_k (q^3;q^3)_k } \equiv q^{2(1-2n)/3}[2n]\frac{(q^3;q^3)_{(2n-1)/3}}{(q^2;q^3)_{(2n-1)/3}}, \end{aligned}$$

where $M=(2n-1)/3$ or $M=n-1$. The congruence (4.2) is equivalent to say that both sides are equal for $a=q^{2n}$ and $a=q^{-2n}$. But this again follows from (2.5) by performing the parameter substitutions $q\mapsto q^3$, $a=q$, $b=q^{1-2n}$, $c=q^{1+2n}$, and $d=q^2$. At last, letting $a=1$ in (4.2), we get (1.8). $\square$

## Proof of Corollaries 1.5 and 1.6

### Proof of Corollary 1.5

Letting $n=p$, where *p* is a prime congruent to $1\pmod {3}$, and $q\rightarrow 1$ in (1.5), we obtain

$$\begin{aligned} \sum _{k=0}^{(p-1)/3} (6k+1)\frac{(\frac{1}{3})_k^3}{k!^3} \equiv p\frac{(\frac{p-1}{3})!}{(\frac{2}{3})_{(p-1)/3}}\pmod {p^3}. \end{aligned}$$

Recall that the *p*-adic Gamma function has the properties: for any *p*-adic integer *x*,

$$\begin{aligned}{} & {} \frac{\Gamma _p(x+1)}{\Gamma _p(x)} ={\left\{ \begin{array}{ll}\displaystyle -x,\quad p\not \mid x,\\ \displaystyle -1,\quad p\mid x, \end{array}\right. }\\{} & {} \quad \Gamma _p(x)\Gamma _p(1-x)=(-1)^{a_0(x)}, \end{aligned}$$

where $a_0(x)\in \{1,2,\ldots ,p\}$ satisfies $a_0(x)\equiv x\pmod {p}$. Let $\Gamma (x)$ be the classical Gamma function. Then

$$\begin{aligned} \frac{(\frac{p-1}{3})!}{(\frac{2}{3})_{(p-1)/3}}&=\frac{\Gamma (\frac{p+2}{3})\Gamma (\frac{2}{3})}{\Gamma (1)\Gamma (\frac{p+1}{3})} =\frac{\Gamma _p(\frac{p+2}{3})\Gamma _p(\frac{2}{3})}{\Gamma _p(1)\Gamma _p(\frac{p+1}{3})}\\&=(-1)^{(2p+1)/3}\frac{\Gamma _p(\frac{p+2}{3})\Gamma _p(\frac{2-p}{3})\Gamma _p(\frac{2}{3})}{\Gamma _p(1)}. \end{aligned}$$

By [17, Theorem 14]), for $p\geqslant 5$, we have

5.1 $$\begin{aligned} \Gamma _p(a+mp)\equiv \Gamma _p(a)+\Gamma _p'(a)mp\pmod {p^2}, \end{aligned}$$

and so $\Gamma _p(\frac{p+2}{3})\Gamma _p(\frac{2-p}{3})\equiv \Gamma _p(\frac{2}{3})^2 \pmod {p^2}$. The proof then follows from the fact $\Gamma _p(1)=(-1)^{(2p+1)/3}=-1$. $\square$

### Proof of Corollary 1.6

Letting $n=p$, where *p* is an odd prime congruent to $2\pmod {3}$, and $q\rightarrow 1$ in (1.8), we obtain

$$\begin{aligned} \sum _{k=0}^{(2p-1)/3} (6k+1)\frac{(\frac{1}{3})_k^3}{k!^3} \equiv 2p\frac{(\frac{2p-1}{3})!}{(\frac{2}{3})_{(2p-1)/3}}\pmod {p^2}. \end{aligned}$$

Further,

$$\begin{aligned} \frac{p(\frac{2p-1}{3})!}{(\frac{2}{3})_{(2p-1)/3}} =p\frac{\Gamma (\frac{2p+2}{3})\Gamma (\frac{2}{3})}{\Gamma (1)\Gamma (\frac{2p+1}{3})} =3\frac{\Gamma _p(\frac{2p+2}{3})\Gamma _p(\frac{2}{3})}{\Gamma _p(1)\Gamma _p(\frac{2p+1}{3})} =3\frac{\Gamma _p(\frac{2p+2}{3})\Gamma _p(\frac{2-2p}{3})\Gamma _p(\frac{2}{3})}{\Gamma _p(1)}, \end{aligned}$$

and by (5.1), $$\Gamma _p(\frac{2p+2}{3})\Gamma _p(\frac{2-2p}{3})\equiv \Gamma _p(\frac{2}{3})^2 \pmod {p^2}$$. $$\square $$

## Some Open Problems

Although the *q*-supercongruence (1.5) is not true modulo [*n*] in general, using the same arguments as in the proof of Theorem 1.1, we can show that, for $n\equiv 1\pmod {3}$ and $n>1$,

6.1 $$\begin{aligned} \sum _{k=0}^{M} [6k+1]\frac{(q;q^3)_k^3}{(q^3;q^3)_k^3} \equiv 0 \pmod {\prod _{\begin{array}{c} j|n,\ j>1\\ j\equiv 1 \bmod 3 \end{array}}\Phi _{j}(q)}, \end{aligned}$$

where $M=(n-1)/3$ or $M=n-1$. Letting $n=p^r$ and $q\rightarrow 1$ in the above *q*-congruence, we obtain the following result: for any prime $p\equiv 1\pmod {3}$ and integer $r\geqslant 1$,

6.2 $$\begin{aligned} \sum _{k=0}^{(p^r-1)/d} (6k+1)\frac{(\frac{1}{3})_k^3}{k!^3} \equiv 0 \pmod {p^r}, \end{aligned}$$

where $d=1,3$. Inspired by Dwork’s work [1] and Swisher’s conjectures [23, (A.3)–(L.3)], we propose the following conjecture on Dwork-type supercongruences, which is a uniform generalization of (1.9) and (6.2).

### Conjecture 6.1

Let $p\equiv 1\pmod {3}$ be a prime and let $r\geqslant 1$. Then

$$\begin{aligned} \sum _{k=0}^{(p^r-1)/d} (6k+1)\frac{(\frac{1}{3})_k^3}{k!^3} \equiv p\Gamma _p(\tfrac{2}{3})^3 \sum _{k=0}^{(p^{r-1}-1)/d} (6k+1)\frac{(\frac{1}{3})_k^3}{k!^3} \pmod {p^{3r}}, \end{aligned}$$

where $$d=1,3$$.

Note that the following Dwork-type supercongruence (see [23, (E.3)] and [4, Conjecture 5.3]) has been proved by the first author and Zudilin [9, Theorem 3.5] by establishing its *q*-analogue:

For any prime $p\equiv 1\pmod {3}$ and integer $r\geqslant 1$,

6.3 $$\begin{aligned} \sum _{k=0}^{(p^r-1)/d} (-1)^k(6k+1)\frac{(\frac{1}{3})_k^3}{k!^3} \equiv p \sum _{k=0}^{(p^{r-1}-1)/d} (-1)^k(6k+1)\frac{(\frac{1}{3})_k^3}{k!^3} \pmod {p^{3r}}, \end{aligned}$$

where $$d=1,3$$.

We believe that the following new *q*-analogue of (6.3), which is also a generalization of Theorem 1.1, should be true.

### Conjecture 6.2

Let $n>1$ be an integer with $n\equiv 1\pmod {6}$ and let $r\geqslant 1$. Then, modulo $[n^r]\prod _{j=1}^r\Phi _{n^j}(q)^2$,

$$\begin{aligned} \sum _{k=0}^{(n^r-1)/d}(-1)^k[6k+1]\frac{(q;q^3)_k^3}{(q^3;q^3)_k^3}&\equiv q^{2(1-n)/3}[n]\dfrac{(-q^3;q^3)_{(n^r-1)/3}(-q^{2n};q^{3n})_{(n^{r-1}-1)/3}}{(-q^2;q^3)_{(n^r-1)/3} (-q^{3n};q^{3n})_{(n^{r-1}-1)/3}}\nonumber \\&\quad \times \sum _{k=0}^{(n^{r-1}-1)/d}(-1)^k[6k+1]_{q^n}\frac{(q^n;q^{3n})_k^3}{(q^{3n};q^{3n})_k^3}, \end{aligned}$$

where $$d=1,3$$.

Likewise, we conjecture a Dwork-type generalization of Theorem 1.2 as follows.

### Conjecture 6.3

Let $n>1$ be an integer with $n\equiv 1\pmod {3}$ and let $r\geqslant 1$. Then, modulo $\prod _{j=1}^r\Phi _{n^j}(q)^3$,

$$\begin{aligned} \sum _{k=0}^{(n^r-1)/d}[6k+1]\frac{(q;q^3)_k^3}{(q^3;q^3)_k^3}&\equiv q^{2(1-n)/3}[n]\dfrac{(q^3;q^3)_{(n^r-1)/3}(q^{2n};q^{3n})_{(n^{r-1}-1)/3}}{(q^2;q^3)_{(n^r-1)/3} (q^{3n};q^{3n})_{(n^{r-1}-1)/3}}\nonumber \\&\quad \times \sum _{k=0}^{(n^{r-1}-1)/d}[6k+1]_{q^n}\frac{(q^n;q^{3n})_k^3}{(q^{3n};q^{3n})_k^3}, \end{aligned}$$

where $$d=1,3$$.

## Data Availability

Data sharing not applicable to this article.
